# A call for action to strengthen stakeholder readiness for ICF data exchange in European health data space: a structured narrative review

**DOI:** 10.3389/fpubh.2026.1786004

**Published:** 2026-04-20

**Authors:** Linda Nieminen, Heidi Anttila, Harri Ketamo, Mauro Zampolini, Andrea Martinuzzi, Vincenzo Della Mea

**Affiliations:** 1 Unit of Physical and Rehabilitation Medicine, Department of Rehabilitation, Wellbeing Services County of Pirkanmaa, Tampere, Finland; 2 Faculty of Medicine and Health Technology, Tampere University, Tampere, Finland; 3 Functioning and Support for Employment Services, Finnish Institute for Health and Welfare, Helsinki, Finland; 4 Headai Ltd., Pori, Finland; 5 Department of Rehabilitation, USL UMBRIA 2, Foligno, Italy; 6 IRCCS E Medea Scientific Institute, Conegliano, Italy; 7 Department of Public Health and Pediatric Sciences, Turin University, Torino, Italy; 8 Department of Mathematics, Computer Science and Physics, University of Udine, Udine, Italy

**Keywords:** health information systems, health policy, information dissemination, international classification of functioning, disability, and health, interoperability

## Abstract

**Background:**

The European Health Data Space (EHDS) regulation introduces a transformative framework for the exchange of health data among EU stakeholders. Among its interoperability measures is the inclusion of the International Classification of Functioning, Disability and Health (ICF) as a standard for documenting functioning in patient summaries and discharge reports. While ICF offers a biopsychosocial lens to complement disease-centric classifications, the availability of interoperable ICF data remains uneven.

**Objective:**

This structured narrative review examines the readiness of EU stakeholders to exchange and utilize ICF data within EHDS. It explores current practices from the data availability and technical infrastructure perspectives, discusses influencing factors such as legislative frameworks and socio-ethical conditions, identifies gaps, and proposes actionable recommendations for the future.

**Methods:**

Given the limited data available on this topic, a structured narrative review was performed, including a structured literature search from five databases. Additionally, targeted searches were performed in policy repositories and institutional sources. The search included documents written in English, Finnish, and Italian, and the study objective defined the scope for the literature search. Documents were analyzed to synthesize contextual information across stakeholders, identify gaps, and gain strategic insights.

**Results:**

In total, 78 studies and gray literature references are discussed in the synthesis. The available evidence on ICF data infrastructures across EU stakeholders reveals significant disparities. Many countries lack standardized EHR support for structured ICF data storage. There is a need to include and map the ICF to international key terminologies and health informatics frameworks to ensure semantic interoperability. Low professional awareness further hinders data availability. Unequal digital literacy and limited citizen empowerment compromise efficient use of ICF. To address these gaps, a three-phase roadmap is proposed: (1) promoting ICF awareness and structured documentation, (2) advancing technical integration through FHIR and ontology development, and (3) aligning policy and governance to support scaling.

**Conclusion:**

Integrating the ICF into EHDS is not merely a technical task; it redefines how health is conceived and measured. By addressing readiness across data, technical, legal, and socio-ethical dimensions, the EU can unlock the full potential of functioning data to improve the well-being of its citizens.

## 1 Introduction

As the European Health Data Space (EHDS) regulation approaches implementation in the coming years, its implications are closely examined across the European Union (EU) stakeholders. EHDS facilitates the use and exchange of health data by harmonizing national health data infrastructures and enabling seamless interoperability among stakeholders across EU countries. EHDS enhances the continuity of care and supports decision-making at both the clinical and system levels by leveraging standardized health data, in compliance with EU legislation, while maintaining the highest standards of privacy and cybersecurity (1). It empowers and facilitates the use of health data for all stakeholders, including citizens and patients, healthcare professionals, providers, researchers, industry and developers, and governmental bodies (2). The EHDS regulation is expected to apply to all European Economic Area (EEA) countries (3).

Under the regulation, data will be transferred between stakeholders for both primary (including patient summaries, e-prescriptions, medical imaging and reports, laboratory results, and discharge reports) and secondary purposes (pseudo/anonymized data). It can be imported from electronic health records (EHR), genetic data repositories and biobanks, research data, and wellness applications (1). To ensure interoperability, exchanged data must follow established standards. In patient summaries and discharge reports, the International Classification of Functioning, Disability, and Health (ICF) is considered the standard for exchanging functioning-related data (4, 5).

The ICF is a comprehensive framework and a standardized language developed by the World Health Organization (WHO) to systematically classify and describe health and health-related domains (6). Unlike traditional disease-centric classifications, such as the International Classification of Diseases (ICD), the ICF adopts a biopsychosocial model that comprises body functions, body structures, activities and participation, and environmental factors. When implemented in health care systems, the ICF has the potential to improve personalized care by supporting assessments in health and social care, chronic disease management, and disability services (7–9). The inclusion of the ICF within the EHDS data exchange can enrich patient profiles by adding health aspects beyond diagnosis, thereby improving care, rehabilitation, and personalized treatment.

There cannot be interoperable ICF data without first ensuring that functioning is assessed, documented, and stored systematically in accordance with available structures. The shared common foundation, which stores entities that can be differentially linearized to form all three WHO reference classifications (ICD, ICF, and ICHI, the newly developed International Classification of Health Interventions), ensures the harmonized use of these structured descriptors across all aspects of health (10). Interoperable, structured ICF data offers many possibilities at the patient, professional, and system levels. For patients, it reduces redundant assessments, supports self-management, and improves continuity of care (11). For professionals, it streamlines workflows, enables timely decisions, and supports evidence-based practice (11, 12). At the system level, it facilitates outcome monitoring, resource allocation, and integrated service delivery through interoperable data (13, 14).

**Figure 1:**
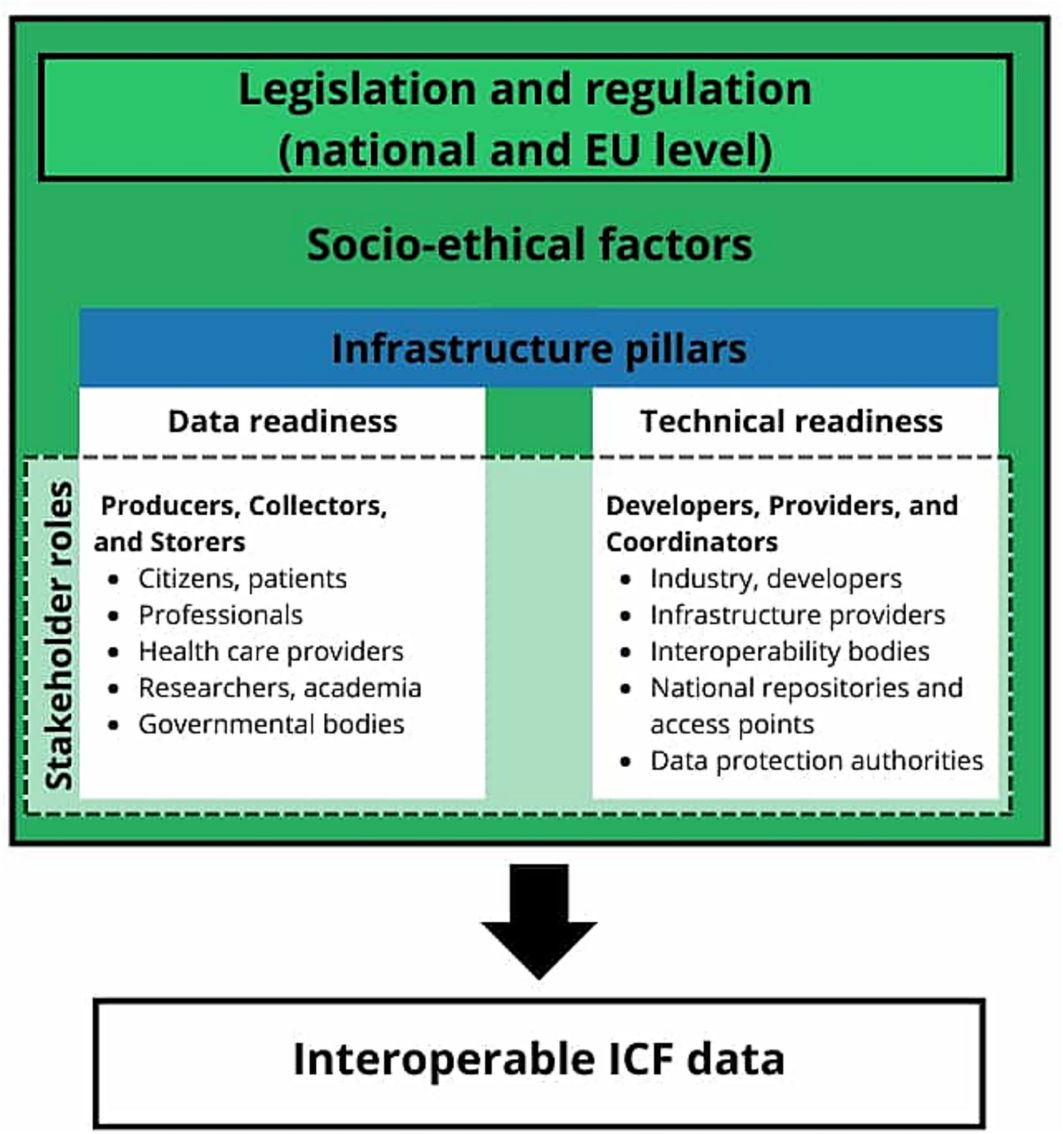
Framework for interoperable ICF data in EHDS: influencing factors, infrastructure pillars, and stakeholder roles.

However, as a representation of the complexity of human functioning, ICF is itself complex and may be difficult to manage without strong technical support. Furthermore, while for other classifications the data source can be confidently limited to specific types of clinical documents or specialties, human functioning data can, in principle, be observed during any encounter or examination, even outside a clinical setting. This makes their storage and retrieval even more complex. The implementation of technical infrastructures to enable recording and storing ICF-coded data in a standardized, interoperable way has been slow, which in turn hinders its possibilities to be exchanged within EHDS. There are gaps in data collection and in technical solutions that would secure seamless interoperability of ICF data. Additionally, it is necessary to consider the influencing legislative and socio-ethical factors when planning data exchange and use (Figure 1). On the other hand, by recognizing the opportunities offered by EHDS, we have the possibility to make a big leap toward large-scale implementation of the ICF, which would bring huge benefits to the well-being and quality of life of individuals.

Our paper examines the readiness of the EHDS stakeholders in EU countries to exchange and use ICF data, while considering legislative and socio-ethical factors. The aim of this paper is to understand the current state of the subject, identify gaps and challenges, and, finally, propose a roadmap to ensure that ICF data will have the potential to be exchanged and used within EHDS.

## 2 Methods

### 2.1 Literature search

Since the evidence base on interoperable ICF data within EHDS remains fragmented, a traditional scoping review was not feasible. Instead, a structured narrative review approach was used. The search strategy followed PRISMA 2020 guidelines (15) to enhance methodological clarity, while allowing the flexibility required for emerging literature. Suitable elements of the PRISMA 2020 framework were applied to ensure transparency of the search and selection process. The applied PRISMA checklist and PRISMA flow diagram are reported in the Appendix 1.

Firstly, targeted searches were performed in databases, policy repositories, EU regulatory documents, and institutional sources relevant to EHDS implementation, especially regarding legislative and socio-ethical factors, and in health organization sources relevant to ICF data use and infrastructures in EU countries (with search words, such as EHDS and legislation/socio-ethical, ICF and “country x”). The expertise of the research group guided the targeted searches. Secondly, a systematic literature search was conducted across databases representing clinical, informatics, policy, and interdisciplinary domains: PubMed/MEDLINE, Scopus, Web of Science, Google Scholar, and CINAHL.

Search terms combined three key concepts: ICF, data or technical enablers, and EHDS. Legislative and socio-ethical aspects were excluded from the Boolean string since these topics are not unique to ICF. The search structure was:

- (“ICF” OR “International Classification of Functioning*” OR “functioning data” OR “functional data” OR “activity limitation” OR “participation restriction”) AND
- (“electronic health record*” OR “EHR” OR “health data” OR “clinical documentation” OR “ontology” OR “terminology” OR “semantic interoperability”) AND
- (“European Health Data Space” OR “EHDS” OR “HealthData@EU” OR “EU health data” OR “Europe*” OR “interoperability” OR “cross-border”).

Given the specific terminology of EHDS and ICF, the search strategy did not include expanded synonym sets or controlled vocabulary. Systematic searches from databases were conducted March 6, 2026. No date restrictions were applied due to the novelty of the EHDS regulation.

### 2.2 Study selection, screening, and data synthesis

Eligible documents included peer-reviewed articles, conference proceedings, EU policy texts, technical reports, web pages, and other gray literature. Materials were included if they addressed

1. Interoperability within EHDS, including legal, technical, or governance frameworks for exchanging structured health data relevant to the ICF.
2. ICF-related data infrastructures and standardization, including efforts to integrate functioning data into health information systems.
3. Data availability and technical enablers, such as semantic mapping or other mechanisms relevant to ICF use in the EU.
4. Professional, organizational, or socio-ethical factors influencing the adoption of structured ICF data.

Documents not addressing ICF, health data infrastructures or results not transferrable to the context of ICF data exchange within EHDS were excluded.

Titles were screened for relevance by one author. The abstracts and full texts were reviewed by two authors to extract information on the above-mentioned topics. Because the goal was thematic understanding rather than exhaustive mapping, no quantitative synthesis was conducted. Qualitative data extraction was conducted to identify the existing practices and determine gaps for future research and development.

Extracted data were analyzed thematically. Findings were grouped into four readiness dimensions defined by authors and supported by the evidence: data availability, technical infrastructures, legislative conditions, and socio-ethical factors.

After removing duplicates, 341 titles and abstracts were assessed for eligibility, and finally, 78 studies and gray literature references were included in the narrative review, which is presented in the next chapter.

## 3 Current state and challenges

### 3.1 Data readiness: instruments for data collection, storage, and practices of using ICF data

Several tools and practices have been developed to support the collection and use of ICF data across clinical, research, and policy contexts. The ICF Core Sets are condition-specific selections of ICF categories designed to streamline data collection in clinical settings (16, 17). The WHO ICF Checklist (18) provides clinicians with a tool for recording functioning and disability using a simplified subset of ICF categories. The ICF framework has been used in the development of questionnaires, such as the generic questionnaire WHODAS 2.0 (19) and those designed to collect data from specific groups, such as children (20) or people with hearing difficulties (21). To streamline ICF code searches, the ICF coding tool serves as an interactive online search engine with both regular and flexible search options (22). The harmonization of standardized data collection is facilitated by the ICF linking rules (23), which have resulted in the development of approaches in, for instance, mental health (24). Additionally, the mICF (ICanFunction) mobile application represented a person-centered digital tool that allowed individuals to self-report functioning data, facilitating personalized care (25).

Readiness to use ICF and its digital availability vary across countries and systems (see Table 1). While some countries, such as Italy, France, Sweden, Finland, Iceland, and Germany, have made progress in embedding the ICF into national or regional workflows, many still rely on conceptual use without standardized coding (26). Broader adoption is still emerging.

**Table 1:** ICF data use and supporting infrastructures across EU and EEA countries (26, 29, 35, 93–110).

Country	Main areas of use	Documentation practices and EHR integration	National repositories, legislation	Interoperability efforts	Training and support
Austria	Neurorehabilitation, dietetics	Used mainly at unit/project level; no national EHR solution	None reported	None reported	Introduced in academic curricula
Belgium	Research in social participation	None reported	None reported	No evidence of ICF data interoperability, high eHealth maturity	ICF Practical Toolbox provided by Rehabilitation International Belgium
Bulgaria	Individual needs assessment (conceptual use), Children with special educational needs	None reported	Endorsement by legislation as a concept (persons with disabilities act)	At recommendation stage	Official Bulgarian translation of ICF with guidance documentation
Czechia	Rehabilitation, social services	Not routinely used	None reported	None reported	ICF e-learning tool available in Czech
Denmark	ICF as a framework in health and social care, neurorehabilitation, assessment of outcomes	Not routinely used	No repositories reported; Used as a general framework in policies	No evidence of ICF data interoperability, high eHealth maturity	ICF e-learning tool is available in Danish
Finland	Broad use in rehabilitation and across health and social care	ICF-based documents, structured data model, FHIR profile development	Termeta metadata and code repository; Multiple social and health acts mandate assessment of functioning	FHIR profiles and semantic interoperability; phased FHIR implementation during 2025–26	ICF e-learning tool is available in Finnish. Manuals and training for structured documentation provided by THL and ICF educators.
France	Disability assessment (GEVA tool), research, population surveys	Limited in routine care; GEVA tool used; no EHR integration	National disability survey	Focus on consent and data protection	ICF e-learning tool available in French, GEVA training for professionals
Germany	Rehabilitation, disability services, needs assessment	Partial and regionally varied assessment tools; EHR integration fragmented	BTHG and social law make ICF the basis for needs assessment	Digital transformation in progress	Widely required and offered for social and health professionals
Hungary	Population level disability reporting including ICF concept	None reported	Census database	None reported	None reported
Iceland	Work ability, disability and rehabilitation needs assessment, vocational rehabilitation (VIRK)	Routine ICF coding in EHRs, not yet mandatory or fully implemented	ICF-based integrated expert disability assessment (from 09/2025)	Not ongoing efforts, but ICF supports semantic interoperability of functioning data across services	ICF materials used in professional education; VIRK and professional associations provide ICF-based training and guidance
Italy	Disability evaluation, rehabilitation planning, school inclusion for children with disability	Regionally implemented tools; EHR and registry-related ICF use is developing	Istat National repository regulated by MoH, Institutional repositories of EHR School records; framework law on disability	ICF used in ISTAT statistics, national infrastructure supports interoperability	ICF training for teachers and professionals
The Netherlands	Rehabilitation (assessments of functioning and need for aids), nursing, and research	Not routinely used, no evidence of EHR integration	None reported	None reported	ICF e-learning tool is available in Dutch, mandatory training for some professionals, introduced in academic curricula
Poland	Rehabilitation, physiotherapy	Pilot studies ongoing, ICF Rehabilitation Set tested; no EHR integration	None reported	No evidence of ICF data interoperability, high eHealth maturity	ICF e-learning tool is available in Polish
Romania	Disability assessment for children (more conceptual use)	No systemic approach for documentation, EHR integration not available	Legislative endorsement; no repositories available	None reported	Limited training so far, efforts for wider support
Slovakia	Social service needs assessment	None reported	Legislative endorsement on functioning (not explicitly ICF); no repositories	None reported	None reported
Slovenia	Vocational rehabilitation and disability assessment	None reported	Legislative endorsement; no repositories	None reported	None reported
Spain	Exploratory use in rehabilitation and physiotherapy	Not standardized, Academic and pilot contexts; no evidence of EHR integration	None reported	None reported	ICF e-learning tool is available in Spanish
Sweden	Social care, elderly care, psychiatric services	Implemented in regional EHRs; Structured documentation supported by Socialstyrelsen	Regional repositories in social care	Regional integration	ICF e-learning tool is available in Swedish. Manuals and training provided by Socialstyrelsen

BTHG, Bundesteilhabegesetz, Federal Participation Act (Germany); EHR, Electronic Health Record; FHIR, Fast Healthcare Interoperability Resources; GEVA Tool, Guide d’évaluation multidimensionnelle, multidimensional assessment guide (France); ICF, International Classification of Functioning, Disability and Health; ICF Rehabilitation Set, A subset of ICF categories (Poland); ISTAT, Istituto Nazionale di Statistica, the Italian National Institute of Statistics; Kanta Services, Finland’s national digital health infrastructure; MoH, Ministry of Health (Italy); Socialstyrelsen, Swedish National Board of Health and Welfare; Termeta, Metadata Repository (Finland); THL, Terveyden ja hyvinvoinnin laitos, Finnish Institute for Health and Welfare; VIRK, Icelandic Vocational Rehabilitation Fund. None reported, No supportive evidence was found.

Ontology-based initiatives have demonstrated the feasibility of formally representing ICF concepts. An early example is the Italian ePlanning project (27), which developed an ontology-driven system to support individualized education plans using the ICF-CY framework. This work was built upon a formal ICF-CY OWL ontology (28). The resulting SOFIA platform operationalized these ontological models for real-world use, enabling semi-automatic structuring of functioning information in educational and rehabilitation contexts.

Maritz et al. (29) reviewed technical approaches for integrating ICF into EHRs, including combining it with existing standards and using natural language processing to select codes. The authors emphasized the need for developing an ICF ontology and linking it to clinical terminologies like SNOMED-CT, although another study by Gongolo (30) suggested that especially activities and participation related to ICF categories are semantically and often lexically different from the concepts of SNOMED-CT. Furthermore, integration of ICF into international health informatics standards remains limited. For example, the ICF is not currently included among the standard vocabularies in the Observational Health Data Sciences and Informatics (OHDSI) framework, representing a significant gap in structured ICF data storage and interoperability.

Fortunately, there are ongoing efforts to harmonize ICF to multiple classifications. For example, The Belgian SNOClass initiative (31) aims to map SNOMED-CT to ICD, ICHI and ICF, providing evidence of multi-classification harmonization efforts. Recent developments such as the ICPC-3 revision (32) demonstrate a conceptual shift toward integrating functioning into primary care classifications using the ICF framework. Although not yet semantically harmonized (33), this indicates a broader trend of converging classification systems around the ICF framework.

At the European level, semantic interoperability topics are addressed through working-level discussions within the eHealth Network, which support shared approaches to terminology and coding across Member States. Although these activities suggest ongoing collaboration on semantic harmonization, their scope and outputs are not publicly documented.

The level of health professionals’ readiness is essential for ensuring that ICF data is collected and available. Although training programs and documentation guides exist (34, 35), barriers such as low awareness, limited demand for ICF-coded data, and a lack of user-friendly digital tools persist (26). Despite current efforts, further education and system-level support are needed to ensure high competence and consistent use across disciplines. To improve usability and reduce time constraints, documentation systems should support clinical workflows.

Artificial intelligence (AI)-assisted tools have shown potential in enhancing documentation efficiency and accuracy by suggesting relevant codes, identifying gaps, and supporting clinical reasoning (36). Such technologies can reduce cognitive load and improve data quality, especially in multidisciplinary settings (37).

Despite promising national initiatives and emerging digital tools, global integration of the ICF into daily documentation workflows, EHRs and national databases remains uneven, with gaps in harmonized standards and professional readiness posing ongoing challenges.

### 3.2 Technical readiness: development and coordination of interoperability and technological solutions

To facilitate seamless cross-border exchange of ICF data and other health data for primary and secondary purposes within EHDS, national health data infrastructures must meet a comprehensive set of requirements. As a general requirement, systems must ensure high performance, patient safety, and uphold individuals’ rights to access and control their data. Operational integrity and compatibility with other digital health products are also mandated for both primary (e.g., EHR systems) and secondary use (including secure processing environments). Compliance with EU legislation is mandatory to ensure security and cybersecurity. Mechanisms for user identification, authentication, authorization, and event logging are required. Systems must support structured data entry in compliance with established standards (such as Fast Healthcare Interoperability Resources, FHIR) and align also with other specifications defined in the European Electronic Health Record Exchange Format (EEHRxF) (38). All health data systems must comply with EHDS market surveillance mechanisms, which include mandatory testing, registration, and ongoing monitoring to ensure interoperability and data security (39).

The EEHRxF prioritizes six categories, including patient summaries and discharge reports, which include the ICF as a standard for exchanging functioning data (4, 5). The European Commission funds and coordinates a number of projects that will ease the adoption of EEHRxF, including Xt-EHR for primary use, XpanDH for ecosystem building, and Xshare for data sharing (40).

Fast Healthcare Interoperability Resources (FHIR) profiling is essential for enabling structured and interoperable exchange of ICF data within EHDS. The WHO-FIC (Family of International Classifications) network has emphasized the importance of FHIR profiles in operationalizing reference classifications (ICD, ICF, ICHI) within EHDS (41). Concurrently, the PACIO Project has developed FHIR-based models to represent functioning and post-acute care data. In PACIO’s pilot, patient data was exchanged across 12 systems using standardized HL7 FHIR resources, with ICF serving as the semantic framework for describing functioning and participation throughout the care continuum (42). These initiatives provide a valuable foundation for developing an approach to EHDS-aligned health information systems.

Several national systems are already taking steps toward EHDS technical compliance, thus enabling interoperable use of ICF data. In Finland, the Kanta services are implementing functioning data across health and social care, with HL7 FHIR profiling underway and integration into the national data repositories planned to start in 2026 (43). Italy’s electronic health data repository (Fascicolo Sanitario Elettronico, FSE 2.0), while regionally managed, is being expanded through EHDS to include broader health and social data, creating a pathway for ICF data. Although ICF is not yet explicitly included, the system’s alignment with EHDS interoperability goals suggests readiness for such integration (44). In Iceland (a European Economic Area country), the Heilsuvera platform provides centralized access to structured health data (such as prescriptions) and services (such as appointment booking), and while ICF data is not yet documented, the platform’s modular architecture and adherence to EU digital health standards position it well for future adaptation (45). These examples illustrate how national infrastructures can evolve to meet EHDS technical requirements while supporting both primary and secondary use of structured functioning data.

Artificial Intelligence is increasingly used to automate the standardization of health and functioning data, including ICF data. Different studies (12, 37, 46–50) demonstrate that AI systems using Natural Language Processing (NLP) techniques can extract, classify, and validate functioning data from diverse sources and contexts. These systems have the potential to scale across professional domains and wellness applications, reduce manual workflows, reduce inconsistencies and enhance the secure interoperability of ICF data. As EHDS develops, AI-powered curation tools will be essential for ensuring structured, high-quality ICF data exchange across borders. As a new emerging technology, agentic AI will bring new possibilities for data interoperability and the integration of novel data sources (51). While the possibilities are remarkable, agentic AI introduces a new layer of cybersecurity challenges that should be studied thoroughly (52).

Achieving technical readiness among EU stakeholders requires coordinated implementation of the above-mentioned requirements. WHO-FIC interoperability hub provides a concrete guidance for integrating WHO classifications into interoperable health systems (53). WHO-FIC Foundation Component already provides a shared ontological backbone for ICD-11, ICF and ICHI, but it does not in itself constitute a domain-specific operational ontology for EHDS-level functioning data exchange (54, 55). It must be noted that earlier ICF ontologies are not harmonized to current requirements nor to EU-wide interoperability (53). Further efforts are needed to align all national infrastructures to these requirements, ensure semantic interoperability across diverse data sources, and enable seamless cross-border exchange while maintaining the highest security standards.

For secondary data use, multiple health data access bodies (HDABs) may exist within one country. Establishing governance structures for metadata stewardship and system certification for secure processing environments are essential for the effective and secure data exchange (56).

Although several national systems are progressing toward technical EHDS compliance, the integration of ICF data remains limited. ICF is not yet part of key international standards, and semantic interoperability is insufficient. Many EHR systems lack support for structured ICF data, and AI tools for automation are still emerging. Without efforts to align infrastructures and ensure interoperability, the potential of ICF within the EHDS cannot be fully realized.

### 3.3 Legislative readiness: legal frameworks and conditions for cross-border data use

EU-level and national legislations serve as primary external forces shaping the conditions under which structured health data, including but not limited to ICF data, is collected, exchanged, and utilized within EHDS. The EHDS Regulation, in conjunction with the General Data Protection Regulation (GDPR) and the Data Act, establishes a comprehensive legal framework that governs both primary and secondary uses of health data across EU Member States (1). While the GDPR remains the foundational regulation for personal data protection, the EHDS regulation introduces sector-specific provisions for healthcare, such as enhanced data portability, harmonized interoperability standards, and the establishment of Health Data Access Bodies (HDABs) to oversee secondary use requests (57).

The Data Act supplements these regulations by defining rights and obligations concerning data generated by connected health technologies, such as wearables and medical devices. It strengthens user control and mandates fair access to data, thereby facilitating the integration of ICF data from multiple sources (58).

The EHDS Regulation has a dual framework for health data use, consisting of different provisions for primary and secondary use. Currently, Member States differ in their capacity to implement them effectively. Countries like Estonia and Finland have developed integrated digital health infrastructures that align well with these goals (14, 59), whereas others, such as Germany and Italy, face challenges due to fragmented systems and regional disparities (60, 61). To support the implementation, the European Commission has launched a series of strategic projects (40). These projects play a critical role in operationalizing the EHDS regulation and ensuring that structured health data exchange, including ICF-based information, is aligned with mandated legislation.

Provisions for secondary use create a legal framework for the reuse of health data in research, public health, and innovation, managed by HDABs. For example, Finland’s Findata offers a ready-made model for this (62), whereas many Member States are still developing the required administrative and technical capacities.

Legislation can play a key role in advancing the use of ICF. A recent example is Italy’s disability law reform, which adopted ICF as the national framework for assessing and supporting people with disabilities. It also updates terminology and establishes a national authority to oversee disability rights and individualized support plans (63). This demonstrates how legislation can drive structured and inclusive use of functioning data.

Legal fragmentation remains a key barrier. While the GDPR provides a common foundation, Member States hold discretion over sensitive health data, leading to different interpretations and requirements that complicate cross-border data exchange and use. Additionally, the collaboration of organizations remains fragmented. Nordic experiences show (64) that even with strong legislation, data exchange often remains hindered by institutional border work between data holders and authorities. Administrative capacity also varies. Health Data Access Bodies are expected to evaluate data access requests for legal, ethical, and technical compliance, including data minimization and secure processing. This requires multidisciplinary expertise and sufficient resources, which are not consistently available (65). Tehdas2, a European Union-funded joint action project, develops guidelines and technical specifications to support the cross-border secondary use of health data and aims to prepare EU countries for this (66).

Effective implementation of EHDS requires more coordinated efforts to harmonize legal frameworks, invest in infrastructure, and strengthen stakeholders’ capacity. Without such alignment, disparities in readiness may hinder the achievement of EHDS objectives and restrict the equitable use of structured health data, including ICF data, across Europe.

### 3.4 Socio-ethical readiness: equity, citizen empowerment, and responsible use of data

The adoption of EHDS must consider multiple socio-ethical factors. These include finding balance with data usability (findable, accessible, interoperable, usable: FAIR principles), safeguarding privacy and security, ensuring equity and access to prevent discrimination and unintended consequences, promoting transparency and explainability in data collection, and supporting the sustainability of digital infrastructure (3, 67).

As the data and technical readiness to exchange and use health data differ between countries, so does the level of health and technology literacy among European citizens. The capacity of individuals to engage with information and technology depends on multiple factors (68). In 2023, approximately 56% of people aged 16 to 74 in the EU possessed at least basic digital skills. The Netherlands (83%) and Finland (82%) had the highest share of people with at least basic digital skills, while Romania (28%) and Bulgaria (36%) recorded the lowest shares. Socio-demographic factors, including age and education level, greatly influenced digital skill levels (69). Regarding AI-supported systems, the AI Act (Article 4) shifts the responsibility for skill development to providers and deployers, who should take measures to ensure, to their best extent, a sufficient level of AI literacy of their staff and users. The EU also underlines the importance of increasing AI literacy among citizens through multiple initiatives (70). Digitally disadvantaged and otherwise vulnerable population groups stand to benefit the most from structured functioning data, yet they are simultaneously at the highest risk of exclusion if digital inequalities are not addressed (71).

In line with the GDPR, citizens have the right to deny consent to use, store, or exchange ICF data, and not using these opportunities should not be sanctioned or result in a lack of access to health services (GDPR, Article 7, Conditions for consent) (72). At the same time, citizens need to be informed and empowered so that no possible benefits to promote their health are undermined. Citizens should be aware of the intended purposes for using ICF data within EHDS. Health literacy on ICF and the importance of functioning data should be promoted.

The GDPR recognizes the individual as the primary owner of the data, but the data controller determines the purpose and means of processing the data (e.g., hospital or national data registry). My Data, a non-profit association, envisions that within EHDS, the approach to implementation should be individual-centric and not population-centric, as proposed at the moment (73, 74). Whatever the means of sharing data, trust in the shared data across the EU should be established through patient/citizen portals, where individuals can see, share, and correct any health information about themselves, including ICF data. This could, with robust legislation (e.g., the AI act, Article 5) (75) and surveillance, minimize the risks posed by datafication (76). It must be noted that approximately 65% of countries enable citizens to exercise granular access control over which specific health information can be shared and with whom (77), highlighting persistent variation in individual data sovereignty across Europe.

Although ICF does not include personal factors, the functioning involves factors related to disabilities, everyday activities, and benefits related to one’s income level. Additionally, there are cultural differences affecting activities and participation, as well as environmental factors. With this in mind, diversity and non-discrimination in all phases of data collection, sharing, and AI training have to be taken into account (78). Multinational and multidisciplinary teams are needed to avoid bias (79). It is also essential to incorporate multiple conceptualizations of disability, not only the medical model but also social and biopsychosocial perspectives (80). It is important to note that stigmatization, discrimination and the misuse of data can occur even in the context of anonymous data in secondary use. Harmonization of ethical oversight measurements should be discussed (81). Achieving transparency in collecting and sharing functioning data requires clearly defined data-access mechanisms, formalized data-sharing agreements, and avoiding informal data-sharing channels (82).

The EU is coordinating investments in AI in its Member States as part of its digital strategy (83). However, there might be challenges regarding equitable access to financial resources, and thus, to technical interventions or advancing skills to use them. For example, consortia that are driving digital transformation in countries with higher population densities or with greater technical readiness may be at the forefront of funding opportunities (84). Sustainability of EHDS is not limited to sustainable financing for EHDS primary and secondary data ecosystems but also investing in innovation and fostering of skills and talents (67), to name a few. Furthermore, the process of data transfer imposes a considerable burden on the environment, given the continuous growth in computing demands. Balance between deletion or storage of curated datasets must be reflected (85).

As ICF has faced difficulties in its technical implementation, the same might be ahead when using ICF in the context of EHDS. The benefits of ICF (comprehensiveness, holistic approach, versatility) also present implementation challenges. Other data standards, such as ICD, are already embedded in many health care systems, and thus more easily exchanged between different data holders. However, ICF addresses the socio-ethical challenges posed by many other standards: it can have an impact on all levels of society by concentrating on general aspects everyday life rather than, for example, particular diseases (76).

Socio-ethical readiness for ICF data exchange within EHDS is challenged by unequal digital literacy, limited public awareness, and concerns regarding privacy, equity, and data bias. Cultural diversity and trust in data use must be addressed through inclusive, transparent, and citizen-centric strategies. Investments in education, inclusive design, and strong security are essential to realizing the benefits of ICF data across Europe.

## 4 The roadmap for ICF data availability in EHDS

Ensuring ICF data availability in EHDS requires a structured roadmap that addresses the identified gaps in data readiness and technical infrastructure, while also ensuring legislative alignment and consideration of socio-ethical factors. Ethical principles should be integrated throughout all phases. Citizen-centric interfaces should be prioritized by developing transparent portals that allow individuals to view, manage, and understand their ICF data. Investments in equitable access are essential to reduce discrimination. Artificial Intelligence systems used for documentation should be explainable, inclusive, and free from bias. The use of ICF data should reflect cultural and contextual diversity. Guiding principles such as FAIR, non-discrimination, and sustainability should inform all phases of implementation. Regular ethical impact assessments should be implemented.

### 4.1 Phase 1: promote ICF awareness, capacity building and data structuring

The first phase aims to enhance professional and public readiness for ICF data utilization. Establishing a multinational, multidisciplinary working group is recommended to advance the development of a common ICF data model within EHDS, including guidelines for the collection and documentation of functioning data. Structured documentation practices should be promoted throughout health and social care workflows, supported by targeted training programs and digital tools. The dissemination of AI-assisted solutions is needed to reduce the manual work associated with ICF coding. Practical measures to improve data availability include deploying AI-supported documentation assistants, integrating ICF coding building blocks into existing EHR systems and citizen data portals, and developing user-friendly interfaces to assist professionals in selecting relevant ICF categories during routine workflows. Public awareness campaigns should be launched to improve health and digital literacy, ensuring citizens understand the relevance of functioning data in their care.

### 4.2 Phase 2: promote technical integration and interoperability

The second phase addresses technical barriers to ICF data exchange. National EHR systems should be adapted to support structured ICF data using HL7 FHIR profiles and comply with all other specifications outlined in the European Electronic Health Record Exchange Format (EEHRxF), including requirements for system performance, security, authentication, metadata management, and semantic interoperability. Fast Healthcare Interoperability Resources (FHIR) profiling is central in enabling structured and interoperable exchange of functioning data, particularly in patient summaries and discharge reports where ICF is defined as a standard. Traceability of ICF data must be ensured (where, when, how, and by whom data was collected) so that the data can be effectively used for secondary purposes.

The WHO-FIC guidance on FHIR profile implementation and data interoperability in general (41, 53), the PACIO Project outcomes (42), and Finland’s structured data models for functioning (43) offer concrete examples of technical integration of ICF into EHDS-compliant systems. Pilot projects should be conducted to test cross-border exchange of ICF data.

Semantic interoperability should be strengthened by further developing ICF ontologies and linkages to ICD, ICHI, and international terminologies such as SNOMED-CT. Inclusion of ICF into international health informatics frameworks, such as OHDSI, is essential to facilitate secondary use and research.

### 4.3 Phase 3: promote policy alignment, governance and scaling

Policy alignment applies broadly to all health data exchange and consists of harmonizing national legislation with the EHDS regulation. From a governance perspective, health data access bodies (HDABs) should be equipped with the capacity to evaluate requests related to ICF data. The inclusion of ICF data in secondary use taxonomy requires the definition of its intended purposes and authorized users. ICF data availability and quality on healthcare and social services should be continuously monitored and the impact evaluated to assess the effectiveness and enhance knowledge management. Best practices that improve ICF data availability should be extended to all EU member states. The transition from pilots to broader deployment should be funded and supported by the EU. Surveys should be used to assess citizen and patient satisfaction, and co-creation among stakeholders should be promoted.

To facilitate the implementation of the proposed roadmap, a comprehensive checklist covering all key focus areas is provided (Appendix 2). While the checklist aligns specific focus areas with relevant stakeholder groups, effective implementation of the roadmap requires strong collaboration in practice.

## 5 Discussion

The inclusion of functioning data within EHDS is not merely a technical enhancement. It reflects a fundamental shift in how health is conceptualized and measured. Traditionally, health systems have relied on mortality and morbidity as core indicators. Functioning, by integrating biological health with lived health, can serve as the third core indicator of health. This framing highlights the value of health for both individual well-being and societal welfare and can drive investment and progress toward the United Nations Sustainable Development Goal “Ensuring healthy lives and promote well-being for all at all ages” (SDG 3) (86). Thus, functioning data, coded through the ICF, provides a more complete picture of health system performance and enables monitoring across all five health strategies recognized by the WHO’s universal health coverage initiative: promotion, prevention, treatment, rehabilitation, and palliative care (87, 88).

Despite the strategic inclusion of ICF in the EHDS framework, several key challenges must be addressed to ensure successful implementation. The roadmap presented in this paper addresses these challenges through a three-phased program that should be used as an iterative cycle of development. Iteration could be triggered when new standards or technologies, governance requirements, or semantic data domains require reassessment of workflows and stakeholder roles. Such cycle should be governed at EU level, possibly under the eHealth Network or a dedicated ICF task force within the EHDS governance structure, with national focal points responsible for monitoring implementation at the Member State level.

Strengths of the current roadmap include the alignment with WHO’s comprehensive health strategies and the use of a standardized classification that enables cross-sectoral and international comparability. The EHDS initiative benefits from strong political momentum, while the ICF framework provides a standardized basis for harnessing the growing recognition of the importance of functioning data in health systems.

Weaknesses to implement the suggested roadmap include limited awareness among EU stakeholders regarding the ICF, fragmented data infrastructures across member states, and the lack of integration between clinical and administrative data systems. A significant limitation is the scarcity of ICF implementation data. Even in contexts where the ICF has been formally endorsed, the evidence on its utility, data quality, and impact on clinical outcomes are limited. This gap makes it difficult to strengthen the evidence base needed to support large-scale ICF adoption within EHDS. Addressing this limitation requires dedicated efforts to collect implementation data from ongoing pilot projects, establish benchmarking frameworks for ICF data quality, and promote the publication of implementation experiences across Member States.

The roadmap presents several opportunities, such as the potential to improve equity in health service delivery, enhance rehabilitation planning, and support wellbeing of individuals through better data-driven insights. The integration of functioning data also opens possibilities for research and innovation.

Implementation of the roadmap faces systemic and external threats that affect all health data exchanged within EHDS, not just ICF data. Data privacy concerns may delay progress or reduce public trust. Uneven digital capacity across EU regions could exacerbate health inequities, while limited policy commitment and funding could hinder long-term integration. WHO analyses (89) highlight that strong national data governance frameworks are essential for ensuring the quality, consistency and trustworthy use of health data. Without clear governance and sustainable investment, ICF data may remain underused despite its strategic inclusion in EHDS.

Since the roadmap was based on narrative synthesis, it is limited by the heterogeneity of the evidence base and scarcity of empirical ICF implementation data, which limits the strength of certain conclusions. Targeted searches may have missed country-specific gray literature or unpublished initiatives. Countries with unsuccessful or discontinued ICF projects rarely publish results, which skews the evidence landscape. Given the expertise of researchers, there is a possibility of bias in targeted searches.

Despite these limitations, we can argue that structured ICF-based data enhances cost-effectiveness by reducing redundant assessments, enabling automated data exchange, and supporting outcome monitoring without the need for separate data collections. Evidence from a cost–benefit analyses demonstrate that investments in ICF data architecture and distribution generate benefits as early as the second year, and all investments are fully covered within less than a decade through efficiency gains, improved resource allocation, and greater equity in service delivery (90).

The maturity of EU stakeholders in deploying EHDS requirements, let alone in exchanging interoperable ICF data, varies considerably across Member States. Some countries already possess robust infrastructures and detailed deployment plans for ICF data exchange, whereas others are still in the initial stages of establishing basic EHR system infrastructure. Those at the forefront should prioritize accessible and transparent documentation of their efforts, enabling other stakeholders to apply these lessons and accelerate their own development.

A viable approach to implementing all recommendations outlined in the roadmap is to conceptualize the process through implementation frameworks. Theoretical frameworks can help us understand facilitators and barriers to implementation and inform strategies that ensure successful dissemination. The context can be analyzed using the Consolidated Framework for Implementation Research (CFIR) (91), which provides a structured basis for strategy development. Additionally, ERIC strategies (Expert Recommendations for Implementing Change) provide a taxonomy of concrete actions designed to enhance adoption, implementation, sustainment, and scale-up of practices (92). Applying these strategies can also enable consistent reporting and facilitate comparison of implementation approaches among EU stakeholders. Adopting a more practical approach, EIT Health Think Thank report (67) shares key findings on stakeholder current capacity, and best practices for EHDS implementation.

When successfully implemented, ICF data can significantly enhance the quality and equity of health and social care across Europe. It enables personalized care planning, supports multidisciplinary collaboration, and improves continuity of care. At the system level, functioning data can inform resource allocation, policy development, and service evaluation. For citizens, it offers a more holistic representation of health, empowering individuals to participate actively in their care and navigate services more effectively.

The roadmap presented in this paper provides a structured approach to advancing ICF data availability within EHDS. However, its successful implementation requires coordinated efforts among EU institutions, national governments, and stakeholder groups. Investments in AI-assisted documentation tools, citizen-centric data portals, and inclusive training programs are critical.

## 6 Conclusion

Integrating the ICF within EHDS represents a transformative opportunity for public health in Europe. Realizing this vision requires immediate and coordinated actions to strengthen data infrastructures and interoperability, ensuring that ICF data becomes a cornerstone of sustainable, data-driven health systems.
