# Supplementary material for: A call for action to strengthen stakeholder readiness for ICF data exchange in European health data space: a structured narrative review
Source: Front Public Health. 2026 Apr 20;14:1786004. doi: 10.3389/fpubh.2026.1786004 (PMC13136167; doi:10.3389/fpubh.2026.1786004)
Supplement: Supplementary file 2 [file Table_1.docx]

# Checklist for stakeholders to implement the ICF data availability roadmap in EHDS

| Stakeholder Group | Implementation Focus | Checklist Item | Status (Yes / No / In Progress) |
| --- | --- | --- | --- |
| Citizens and patients (advocacy groups) | Awareness & empowerment | Citizens are informed about the purpose and benefits of ICF data in EHDS. |  |
|  | Data access | Individuals have access to view, manage, and correct their ICF data via digital portals. |  |
|  | Literacy & inclusion | Initiatives are in place to improve health and digital literacy related to functioning. |  |
| Healthcare professionals and providers | Documentation practices | Structured ICF documentation is integrated into clinical and social care workflows. |  |
|  | Training & support | Professionals are trained to assess, document, and interpret ICF data. |  |
| Research and academia | Secondary use & standards | ICF data is accessible for research under EHDS secondary use provisions. |  |
|  | Methodological development | ICF ontologies and mappings to other terminologies (e.g. SNOMED-CT) are being developed. |  |
| Industry and developers | Technical integration | EHR systems and digital tools are compliant with EEHRxF for ICF data. |  |
|  | Innovation | AI and semantic technologies are used to enhance ICF data interoperability. |  |
|  | Market surveillance | Systems are registered and tested under EHDS market surveillance mechanisms. |  |
| Administrative and regulatory bodies | Legal alignment | National legislation is harmonized with EHDS, GDPR, and the Data Act |  |
|  | Governance | Health Data Access Bodies are equipped to handle ICF-related data requests. |  |
|  | Monitoring & evaluation | Mechanisms are in place to monitor the impact of ICF data integration on health systems. |  |
